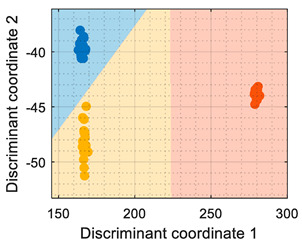# Classification of Three Volatiles Using a Single-Type eNose with Detailed Class-Map Visualization

**DOI:** 10.3390/s22145262

**Published:** 2022-07-14

**Authors:** Jordi Palacín, Elena Rubies, Eduard Clotet

**Affiliations:** Robotics Laboratory, Universitat de Lleida, Jaume II, 69, 25001 Lleida, Spain; helenarubies@gmail.com (E.R.); eduard.clotet@udl.cat (E.C.)

**Keywords:** electronic nose, eNose, array of gas sensors, MOX gas sensors, PCA and LDA analysis

## Abstract

The use of electronic noses (eNoses) as analysis tools are growing in popularity; however, the lack of a comprehensive, visual representation of how the different classes are organized and distributed largely complicates the interpretation of the classification results, thus reducing their practicality. The new contributions of this paper are the assessment of the multivariate classification performance of a custom, low-cost eNose composed of 16 single-type (identical) MOX gas sensors for the classification of three volatiles, along with a proposal to improve the visual interpretation of the classification results by means of generating a detailed 2D class-map representation based on the inverse of the orthogonal linear transformation obtained from a PCA and LDA analysis. The results showed that this single-type eNose implementation was able to perform multivariate classification, while the class-map visualization summarized the learned features and how these features may affect the performance of the classification, simplifying the interpretation and understanding of the eNose results.

## 1. Introduction

An electronic nose (eNose) is an electronic device that uses several gas sensors in order to obtain a characteristic fingerprint of volatiles, odors, or aromas. The raw information provided by the gas sensors is processed with the aim to biomimetically create an artificial nose [1]. The eNose can be considered an alternative device for gas chromatography, being characterized by its low cost, size, and power consumption at the expense of accuracy. An eNose is able to provide a characteristic fingerprint of a volatile, odor, or aroma at concentrations of parts per million (ppm) [2]. However, the precise and accurate detection of volatiles in concentrations on the scale of parts per billion (ppb) presently requires the use of expensive mass spectrometers (MS) or flame ionization detectors (FID), which are usually embedded in large machines that require them to be connected to a high-voltage power supply [3].

Comparatively, an eNose is a relatively simpler gas chromatography device based on the combination of different low-cost gas sensors with different specificities [3]. The main drawback of these low-cost gas sensors are their variability and different sensitivities [4], which can be corrected by performing individual or collective gas sensor calibration [5]. Nevertheless, this variability has been proved to be useful to develop eNoses with multivariate classification performances [6,7].

One of the factors that is currently fostering the use of portable eNose devices is the development and application of compact arrays [8] of low-cost metal oxide (MOX) gas sensors [9], which have known disadvantages of drift in sensitivity and specificity [4,8,10,11,12] that are very difficult to correct or compensate [13], even when using specific signal-processing techniques [5].

Although the first report of an eNose based on MOX gas sensors was provided by Persaud et al. [14] in 1982, it was not until 2002 that the first single-type eNose was proposed by Arnold et al. [15]; it was based on the use of an array of 38 identical custom MOX gas sensors for air-quality monitoring and early fire detection. This eNose demonstrated that a matrix of single-type MOX gas sensors was able to detect different volatile compounds. However, the disadvantage of this proposal was the amount of power required to operate the custom design of the MOX gas sensors. More recently, Bennets [16] proposed in 2012 the use of a matrix of six identical MOX gas sensors to classify two volatiles. The same idea was explored by Burgués et al. [17,18], who in 2018 proposed the use of two eNoses based on 12- and 7-MOX single-type gas sensors to evaluate odor concentrations. In the recent state-of-the-art study of eNoses based on MOX gas sensors presented in [6], the number of gas sensors used in the reported prototypes ranged between 2 and 96 (15.6 on average), while using between 1 and 16 different sensor types (5.5 on average).

Specifically, the analysis of this number of receptors (or gas sensors) used in eNoses is still quite distant from their biological inspiration [19]. An exception was the contribution of Marco et al. [20], who in 2014 proposed the largest eNose reported, which was based on the combination of 96 MOX gas sensors (including 12 different sensor types) and four arrays of up to 4096 chemical gas sensors, with the objective of exploring the development of neuro-bio-inspired computation. More recently, the proposal of the Osmee One eNose [6] was based on the current development of miniature micromachined MOX gas sensors specifically using 16 single-type miniature MOX gas sensors that took advantage of their low thermal inertia, low power consumption, and compact size.

At this moment, most eNose proposals are based on the use of small arrays of gas sensors [21,22] that are optimized for specific applications such as waste management [23,24] or disease detection [25]; however, the visual interpretation of the eNose classification results still needs to be improved, as the importance of portraying the information in a comprehensive way to the human eye [26] may sometimes be critical to improving the classification results and enabling their use in a wider range of scenarios [27].

The new contributions of this paper are the assessment of the Osmee One custom eNose applied to the classification of three volatiles and a proposal to enhance the visual interpretation of the classification results by generating a class-map visualization. The main goal of this second contribution is to enhance the visual interpretation and understanding of the classification results, summarizing the learned features and how they affect the classification, as it was considered that concise data presentation formed the base for subsequent analysis and interpretation. This class-map visualization simplifies the understanding and interpretation of the eNose results, further allowing the detection of conflictive-prone regions and areas of uncertainty that may be critical in derived decision-making procedures.

## 2. Materials and Methods

The materials and methods used in this study were a custom single-type eNose, three target volatiles, a compact gas measurement setup, and different classification methods.

### 2.1. Single-Type eNose: Osmee One

**Figure 1:**
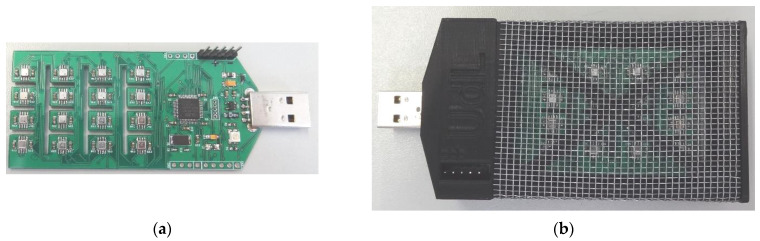
Images of the compact Osmee One eNose used in this paper: (**a**) detail of the electronic board showing the array of sensing devices; (**b**) detail of the case of the eNose using a steel mesh to facilitate air circulation.

The Osmee One eNose used in this paper is a compact and custom proprietary sensor design described in [6] and lately improved in [7]. This eNose uses an array of 16 single-type (identical) miniature micromachined MOX gas sensors embedded in a BME680 sensor device manufactured by Bosch Sensortec. This micromachined sensor was designed to obtain unspecific measurements of the total volatile compounds (TVOC) in the air. The design of this sensor also includes temperature, humidity, and pressure sensors, although the activation of the gas sensor may interfere with the accuracy of these readouts. Figure 1a shows the image of the electronic board of the Osmee One prototype eNose, and Figure 1b shows the case used to protect the devices while facilitating the air circulation. This eNose was designed to operate while plugged into a USB host device, and has a total power consumption of 0.9 W [6] in normal continuous measurement operation.

### 2.2. Three Target Volatiles

In previous works, the Osmee One eNose used in this paper was assessed to classify ethanol and acetone [6,7], and this study assesses the multivariate classification performance using three target volatiles: ethanol, acetone, and butane. These volatiles are ideal candidates when developing portable early gas leak detectors for emergency response scenarios that require compact and portable sensing devices [28,29,30].

### 2.3. Measurement Setup

**Figure 2:**
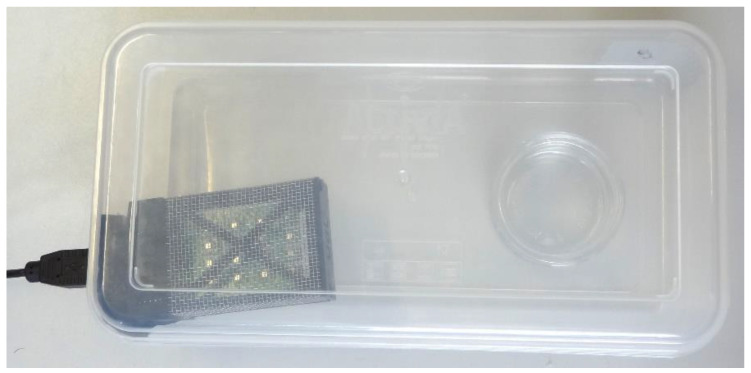
Measurement setup composed of the transparent plastic box, the evaporation glass plate, and the eNose Osmee One.

Figure 2 shows the measurement setup used in this paper, which was the same as the one previously used in [7]. The measurement setup consisted of a small polypropylene (PP) plastic box containing the eNose and a small glass plate on which the liquid form of the volatile substance was deposited for its evaporation. The ethanol and acetone samples were kept in individual polypropylene syringes, with each one containing 1 mL of the target substance; on the other hand, butane was stored in a pressurized canister and could be injected into the plastic box through a dedicated port. The experimental setup used to gather data from the eNose was fully described in [7]. Finally, this measurement setup had the advantage of requiring a relatively short time in order to reach a stable concentration of ethanol and acetone through evaporation.

### 2.4. Methods for Dimensional Reduction and Classification

The methods used for dimensional reduction and classification were based on principal component analysis (PCA) combined with a classifier and linear discriminant analysis (LDA). The application of these methods to the eNose data were fully described in [7]. PCA [31] is an unsupervised method that determines the principal components of a dataset by computing the covariance matrix and the eigenvectors. These principal components allow a reduction in the dimensions of a dataset while maintaining the variability of the clusters it includes [32]. LDA [33] is a supervised statistical method that computes the covariance matrix and the eigenvectors of a dataset while assuming that each cluster has different Gaussian distributions. The discriminant analysis classification model trained with LDA can predict the class of a sample [34]. Despite the differences between PCA and LDA [35], both techniques aim to reduce the dimensionality of the dataset obtained with an eNose [32] by looking for linear combinations of the features that best explain the data. However, PCA focuses on capturing the direction of maximum variation in the dataset (maximizing the variance), while LDA focuses on finding a feature subspace that maximizes the separability between the different clusters composing the dataset (making the clusters as separable as possible).

The methods used for classification after performing a PCA dimensional reduction are: classification-tree (CTree), *k*-nearest neighbors (*k*-NN), and support vector machine (SVM). CTree [36] is a supervised classification method that creates a treelike model of decisions; in this case, defining binary splits for classification. The *k*-NN algorithm [37] is a nonparametric supervised classification method that can predict the class of a sample based on the majority of the class membership of the *k*-closest samples. The SVM [38] was used in this paper as a supervised probabilistic binary classification method that computed a separating hyperplane between two classes. Since SVM is a binary classifier, using it to differentiate between three classes required the combination of three binary SVM models using one-versus-all methods and a winner-takes-all strategy.

The method used for classification in the LDA was based on a discriminant analysis that computed the proximity to the centroid of the clusters of the dataset. In theory, the performance of the LDA classification is expected to be superior, as it makes the clusters as separable as possible; nevertheless, this method assumes that the independent variables of the clusters are normally distributed and have equal variances and covariances across all the clusters, so the performance of the classification can decrease when the correlation between the independent variables increase. For example, in [7], the numeric performance of an LDA classifier applied to two volatile eNose classifications was superior to other PCA-based classification methods.

## 3. Calibration of the eNose with Three Volatiles

**Figure 3:**
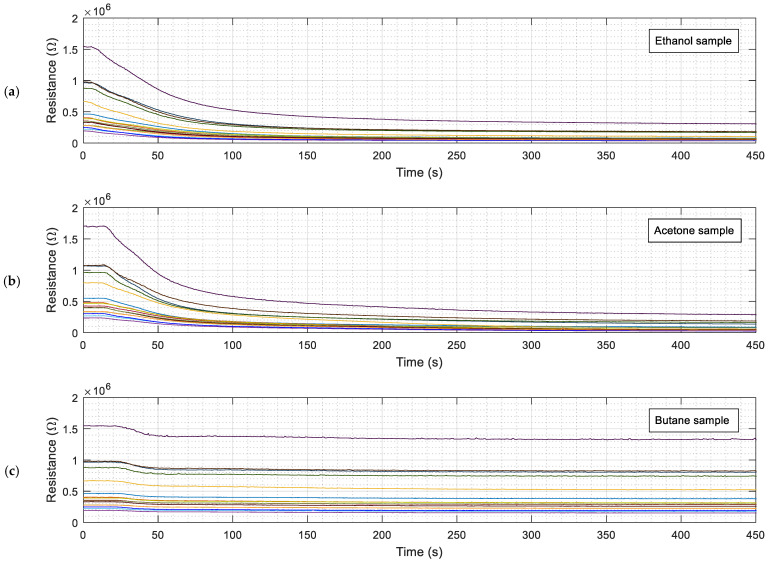
Evolution of the raw resistance of the sensing layer of the 16 MOX gas sensors of the eNose (each one labeled with a specific color) exposed to: (**a**) ethanol; (**b**) acetone; (**c**) butane.

Figure 3 shows an example of the typical evolution of the raw resistance data gathered from the 16 MOX gas sensors of the Osmee One eNose for the cases of liquid ethanol (Figure 3a), acetone (Figure 3b), or butane (Figure 3c) injected in the measurement setup containing clean air. The procedure required to obtain the resistance of the sensing layer of the MOX gas sensors embedded in the 16 BME680 devices used in the eNose was described in [6]. In this study, the evolution of each volatile was measured for 450 s, the equilibrium concentration of the volatiles inside the measurement box was approximately 150 ppm [6], and the box was kept open for at least 60 min between experiments, ensuring that the concentration of the previous volatile was less than 1 ppm [6]. Figure 3 shows that the reaction times of the resistive layer of the MOX gas sensors exposed to ethanol (Figure 3a) and acetone (Figure 3b) were noticeably slower than in the case of butane (Figure 3c); this was because butane was injected as a gas, while ethanol and acetone were injected as liquids (onto the evaporation glass plate) and took time to evaporate. The initial value of the resistance of the MOX gas sensors in clean air was not a constant value, as it exhibited a baseline drift influenced by ambient conditions [39] and aging [12].

## 4. Volatile Classification with Detailed Class-Map Visualization

**Figure 4:**
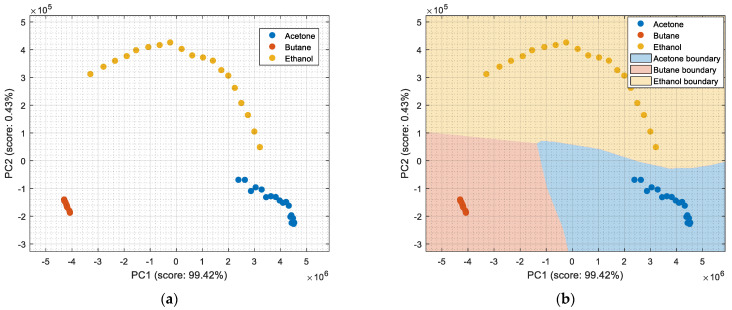
(**a**) Representation of the measures gathered by the eNose in the presence of ethanol (yellow), acetone (blue), and butane (orange) projected in the two principal components defined by the PCA analysis. (**b**) The same representation including a class-map visualization that illustrates the boundaries defined by a *k*-NN classifier applied to the PCA projection results.

This section analyzes the performance of the eNose as multivariate volatile classifier with detailed attention given to the class-map visualization for enhanced direct interpretation. Figure 4a shows the two principal components of the PCA analysis of the calibration dataset obtained in the previous section, expressing the raw resistance data gathered from the 16 MOX gas sensors of the eNose. The calibration data analyzed and displayed was limited to 19 samples of ethanol, 19 samples of acetone, and 19 samples of butane. These sets of 19 samples were obtained by uniformly subsampling the calibration measurements of ethanol, acetone, and butane in the time range of approximately 50 s to 450 s (see Figure 3). This PCA showed that the score of the axis of the first component was 99.42%, the score of the second component was 0.43%, and the score of the third component (not displayed in Figure 4a) was 0.12%, so the first and second components summarized the vast majority of the variance in the calibration dataset corresponding to the three target volatiles analyzed in this paper: ethanol, acetone, and butane. Very similar PCA results were obtained without subsampling the calibration dataset (using approximately 400 samples of each volatile), since it was more important to have the same number of samples from each class than to increase the number of samples.

In order to properly interpret Figure 4a, it must be noted that the scale of the X-axis is almost one order of magnitude higher than the Y-axis, so the data variability on the Y-axis was much lower than on the X axis. Additionally, it must also be noted that the PCA did not process class information, so the labels of each datapoint shown in Figure 4a were manually established before performing the PCA. In this case, the color labels of the datapoints reveal that the three target volatiles were projected across different regions of the two-dimensional surface area defined by the two-principal axis of the PCA, a factor that enabled the development of a feasible classification based on the information represented in this two-dimensional space.

In general, the further classification of unknown gas samples based on the PCA projection is usually represented with performance classification tables or by plotting the unknown gas sample in the PCA projection, as shown in Figure 4a. Since PCA does not provide any sort of classification, assigning the newly projected sample to its corresponding class is left to human interpretation, which is based on using the power of the visual cortex of the brain to visually process and interpret the class membership. This paper aimed to improve human interpretation of the classification process by going one step further by means of including a detailed class-map visualization in the PCA projection. As an example to illustrate this idea, Figure 4b shows the class-map relationship computed with *k*-NN applied to a two-dimensional PCA projection of the calibration points, combined with the knowledge of the class information of all these calibration points. In this case, the *k*-NN classification was based on the class of the *k*-nearest points, so the visual interpretation of the result of a *k*-NN classification is relatively simple for the visual cortex in case of analyzing unknown volatile samples.

**Figure 5:**
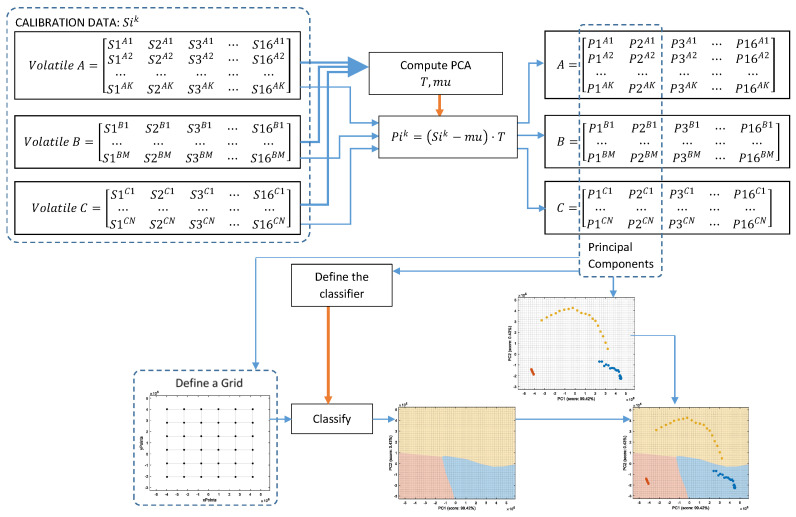
Diagram of the procedure used to compute the class-map representation.

Figure 5 shows the graphical diagram of the complete procedure to generate the images of the classification example shown in Figure 4 which is based on PCA dimensional reduction and on *k*-NN classification. The procedure began with the calibration data containing the data samples of the three volatiles analyzed: A, B, and C, which were joined in order to compute the PCA and estimate the offset to be extracted to each original datapoint of the data, mu (a 16 × 1 vector), and the transformation, T (a 16 × 16 matrix), which must be applied to project the calibration data. The application of this PCA transformation originated a new projected calibration dataset whose axes were sorted according to the variability in the data. Then, the first two elements of the projected calibration data were selected to display the distribution of the clusters in a two-dimensional space, as their combined scores (99.42% and 0.43%, respectively) represented 99.85% of the variability of the classes composing the calibration dataset. In a second step, the information contained in these two principal components was used to define or train a classifier (*k*-NN in this example) and to create a fine or detailed two-dimensional grid with 400 points per axis that are individually classified in order to obtain the visual class-map distribution of this PCA space. Finally, in this example, the projection of the calibration dataset in the two-dimensional PCA space was plotted over the class-map to visually validate the representation. The procedure to compute the LDA class-map followed the same methodology.

The advantage of this class-map visualization is the simplification of the visual interpretation of the class membership of new data gathered from the eNose. Based on our experience, the simplification of the visual interpretation of the eNose classification results has the potential to foster the inclusion of eNoses in emergency applications [40,41] requiring interaction with people [42].

In previous works [6,7], we assessed the performance of different classification strategies based on PCA combined with a and additional classifier, and on LDA applied to classify two volatiles. The preliminary application of these classification strategies to the three volatiles analyzed on this paper yielded almost identical training results for: PCA + CTree, PCA + *k*NN, PCA + SVM, and LDA. Then, the procedure described in Figure 5 allows the enhancement of the visual interpretation of the classification results by incorporating a detailed class-map representation that would help to visually understand the classification results by summarizing the learned features and how those features affect the classification, thus reducing the uncertainty in derived decision-making procedures.

## 5. Assessment of Classification Strategies with Detailed Class-Map Visualization

This section presents the assessment of different multivariate calibration strategies using a detailed class-map visualization in order to simplify the visual interpretation of the performance of the classifiers. Due to the singularity of this eNose proposal based on 16 single-type MOX gas sensors and the novelty of the Osmee One eNose prototype, the assessment performed in this section evaluated all feasible alternatives inspired by the previous Osmee One analysis [6,7] in order to obtain the best multivariate classification alternative for the discrimination of ethanol, acetone, and butane.

As described in the Section 2 of this paper, the classification alternatives assessed were based on: (a) two-dimensional PCA reduction followed by a CTree classification; (b) two-dimensional PCA reduction followed by a *k*-NN classification; (c) two-dimensional PCA reduction followed by an SVM classification applied to the two principal components of the datapoints; and (d) two-dimensional LDA reduction with a discriminant analysis classification. Note that the SVM algorithm can be directly applied to the data gathered from the eNose, but the application of SVM over the two principal components of the PCA provided a visual representation of the classification class-map that simplified its comparison with the other PCA-based classifiers.

For the sake of completeness, the calibration dataset gathered from the eNose was processed as: raw resistance (Table 1), conductance (Table 2), normalized resistance [7] (Table 3), and normalized conductance [7] (Table 4). A first look at the classification rates displayed in Table 1, Table 2, Table 3 and Table 4 confirms that all algorithms yielded similar classification results, which could lead to incorrectly assuming that all classifiers perform similarly. This further underscores the importance of providing a class-map visual representation that complements the classification statistics (displayed in Table 1, Table 2, Table 3 and Table 4 under the “Visual representation” column).

Table 1 shows that the PCA analysis maintained the three volatiles in distinct regions of the PCA projection (although it must be noted that this was not the objective of the PCA, which was to maximize the variability), therefore all PCA-based classification strategies were successful at labeling each observation as the right class. In the case of the PCA + CTree strategy, using binary splits for classification generated a characteristic square-class region distribution; the class-map of the PCA + *k*NN and PCA + SVM resulted in a similar class-map distribution. The comparison of the PCA-based class-maps and the LDA class-map revealed that the specific performance of the LDA was focused on grouping the clusters and making them as separable as possible. Then, according the information displayed in the class-map visualization, the LDA was the most suitable method for the multivariate classification applied to raw resistance data. Conversely, the PCA + CTree strategy was deemed as the least robust solution when compared with the other PCA-based classification methods.

The use of conductance as the input format for the classifiers (Table 2) showed that even though the PCA maximized the variability of the dataset, there was an overlap between the ethanol and acetone samples, creating a region where the ethanol samples were classified as acetone. The difficulty in the interpretation of the dataset as conductance was also highlighted by the LDA analysis, which in its original form (based on computing the distance to the centroid of the clusters) experienced difficulties in this classification. Therefore, the conclusion was that the evaluation of the eNose data as conductance was less reliable for this multivariate classification than its evaluation as raw resistance.

Similarly, Table 3 shows that the PCA analysis of the dataset evaluated as normalized resistance successfully classifies all the training samples; nevertheless, the visual representation of the distribution of clusters showed that some datapoints of the calibration set of ethanol and acetone were projected in a close region; thus, it was to be expected that the performance of these classifiers would decrease, as small sensor drifts may have caused some ethanol and acetone samples to be projected in other class regions. In the case of the LDA classification strategy, the projection of this normalized resistance showed that ethanol and acetone clusters were projected in more distant areas than in the PCA, yet the class-map for the LDA showed that the ethanol and acetone boundaries were quite close to the opposite-class projected training data. This indicated that using the normalized resistance as input data may be unreliable even when using the LDA classification strategy, in which the clusters appeared to be well defined in separate regions of the map.

Finally, Table 4 shows the performance of the classifiers when the eNose data were evaluated using normalized conductance as the input parameter. In this case, the classification class-maps were almost symmetrical than when using normalized resistance, having the same disadvantages.

The conclusion of this section is that the two-dimensional LDA projection of the calibration dataset evaluated as raw resistance provided the best clustering of the calibration data while keeping them as separable as possible, resulting in a well-defined class-map representation. In a previous analysis that focused on classifying two volatiles [7], it was observed that the LDA classification strategy using the raw resistance yielded worse results than those obtained when using the normalized conductance. This contrasted with the results obtained in this work, in which using LDA with raw resistance resulted in one of the best classification strategies. This can be attributed to the fact that the third substance (butane) analyzed in this study increased the complexity of the classification process, affecting the performance of the different classification strategies.

## 6. Validation

**Figure 6:**
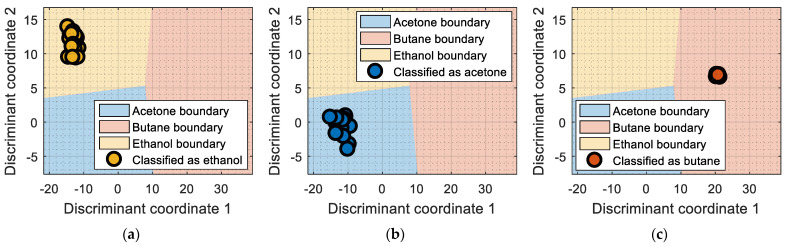
LDA validation results processing the eNose data as raw resistance. The datasets shown contained samples of: (**a**) ethanol; (**b**) acetone; (**c**) butane.

This section presents the validation results of the LDA classifier applied to the raw resistance data gathered from the Osmee One eNose. This validation was performed with a validation dataset containing one measurement performed with ethanol, one with acetone, and one with butane, with each one containing approximately 450 numerical samples in a time range of 450 s. This validation dataset was uniformly subsampled from 50 s to 450 s to obtain 19 samples of ethanol, 19 samples of acetone, and 19 samples of butane. Figure 6 shows the projection of this validation data using the LDA axis obtained during the training. The class-map interpretation displayed in Figure 6 evidences that all the validation samples were correctly classified and contained within the correct volatile region boundaries.

In summary, the comparison of the representation displayed in Figure 6 and of the simpler graphical representation provided in previous papers [6,7] enhanced the correct visual interpretation of the eNose volatile classification in a way that may foster the development of future portable, real-time applications in which human interpretation can be required. In this validation stage, the LDA classifier was evaluated using three different datasets, with each one containing samples of ethanol, acetone, and butane, respectively. During this phase, the classifier obtained an average classification success rate of 100% under laboratory conditions although it is to be expected that the results could worsen when tested in real environments. In the near future, the performance of the Osmee One eNose will be evaluated in uncontrolled environments while operating as an earlier volatile leak detector embedded in a mobile robot. Finally, in [6,7], this single-type Osmee One eNose using the MOX gas sensors embedded in 16 BME680 devices was able to discriminate between ethanol and acetone; while in this study, it was able to differentiate between ethanol, acetone, and butane. These results confirmed the multivariate classification capabilities of the Osmee One eNose based on single-type gas sensors.

## 7. Conclusions

This paper assessed the multivariate classification performance of the Osmee One custom prototype eNose, which is composed of 16 single-type MOX gas sensors embedded in 16 BME680 devices. The eNose was applied to classify three volatiles: ethanol, acetone, and butane, by assessing different classification methods based on a dimensional reduction performed with PCA and LDA: transforming the 16 dimensions provided by the 16 resistance datapoints describing the sensing status of the 16 MOX gas sensors into a simplified, two-dimensional representation that maintained most of the variability of the data samples. The results showed that the data transformation and discriminant analysis performed using LDA was able to successfully classify the three target volatiles. Therefore, the small but inherent variability of 16 single-type MOX gas sensors can provide multisensorial information required to detect volatile diversity. Additionally, this paper proposed a methodology tailored to providing a detailed class-map visualization that is able to graphically summarize the learned classification features and show how these features may affect the performance of the classification. The advantages of using a detailed class-map visualization is the simplification of the graphical interpretation and understanding of the eNose results, a factor that has potential to foster the development of new real-time supervised eNose applications such as emergency evaluation of scenarios.

The conclusion of this paper is that the two-dimensional LDA projection of the raw resistance calibration data provided by the eNose was the most successful in grouping the three volatile classes assessed, making them as separable as possible, while the class-map representation provided a graphical visualization of the classification areas. The validation of the performance of the classifier provided an average classification success rate of 100% under laboratory conditions. These results confirmed the multivariate classifier capabilities of the Osmee One eNose concept composed of 16 single-type MOX gas sensors. However, at this moment, the question regarding how many identical MOX gas sensors are enough to classify two or three volatiles is still open, since the use of 16 MOX gas sensors in this eNose design was established for practical size and power requirements only.

In a future work, the performance of the Osmee One eNose will be evaluated in uncontrolled environments while operating as an earlier volatile leak detector embedded in a mobile robot, and will be applied to detect odors (gas mixtures) in specific practical applications.

## Figures and Tables

**Table 1:** Performance of the classifiers when processing the information gathered from the eNose as raw resistance: representations of true positive (TP) and false positive (FP).

Algorithm	Acetone TP (%)	Acetone FP (%)	Butane TP (%)	Butane FP (%)	Ethanol TP (%)	Ethanol FP (%)	Visual Class-Map Representation
(a) PCA + CTree	100	0	100	0	100	0	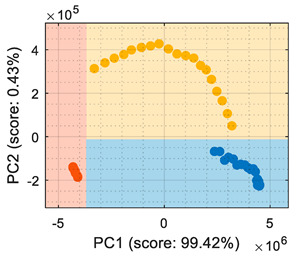
(b) PCA + *k*NN	100	0	100	0	100	0	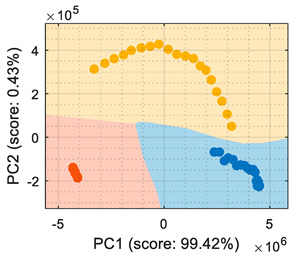
(c) PCA + SVM	100	0	100	0	100	0	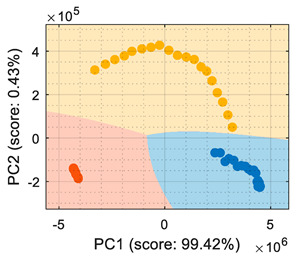
(d) LDA	100	0	100	0	100	0	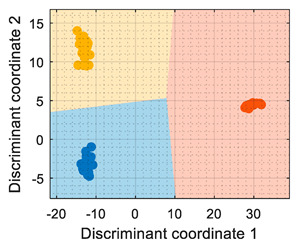

**Table 2:** Performance of the classifiers when processing the information gathered from the eNose as conductance: representations of true positive (TP) and false positive (FP).

Algorithm	Acetone TP (%)	Acetone FP (%)	Butane TP (%)	Butane FP (%)	Ethanol TP (%)	Ethanol FP (%)	Visual Class-Map Representation
(a) PCA + CTree	89.47	0	100	0	100	5.26	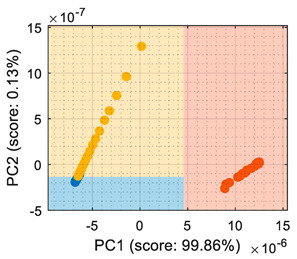
(b) PCA + *k*NN	94.73	5.26	100	0	89.47	2.63	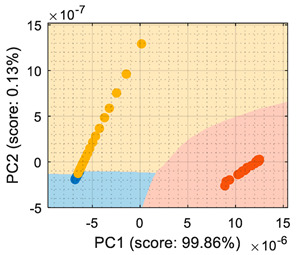
(c) PCA + SVM	100	5.36	100	0	89.47	0	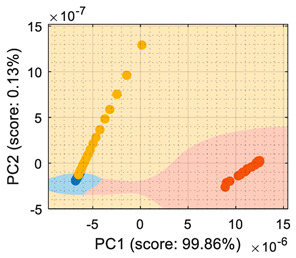
(d) LDA	100	2.63	100	0	94.73	0	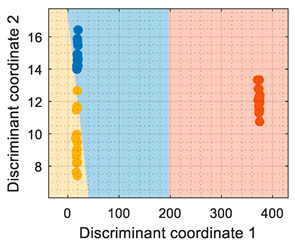

**Table 3:** Performance of the classifiers when processing the information gathered from the eNose as normalized resistance: representations of true positive (TP) and false positive (FP).

Algorithm	Acetone TP (%)	Acetone FP (%)	Butane TP (%)	Butane FP (%)	Ethanol TP (%)	Ethanol FP (%)	Visual Class-Map Representation
(a) PCA + CTree	100	0	100	0	100	0	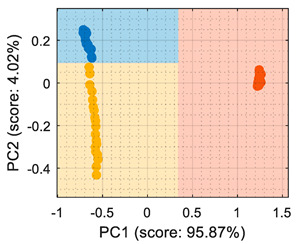
(b) PCA + *k*NN	100	0	100	0	100	0	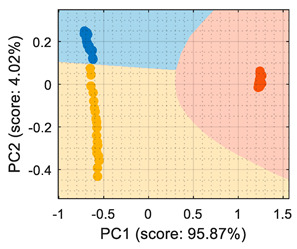
(c) PCA + SVM	100	0	100	0	100	0	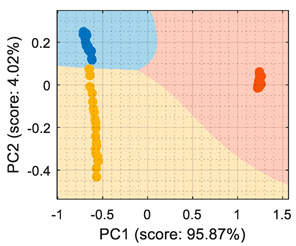
(d) LDA	100	0	100	0	100	0	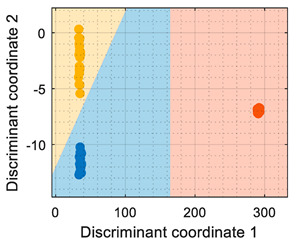

**Table 4:** Performance of the classifiers when processing the information gathered from the eNose as normalized conductance: representations of true positive (TP) and false positive (FP).

Algorithm	Acetone TP (%)	Acetone FP (%)	Butane TP (%)	Butane FP (%)	Ethanol TP (%)	Ethanol FP (%)	Visual Class-Map Representation
(a) PCA + CTree	100	0	100	0	100	0	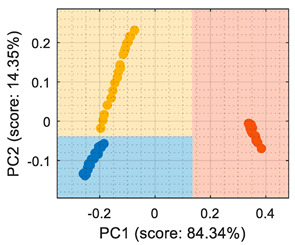
(b) PCA + *k*NN	100	0	100	0	100	0	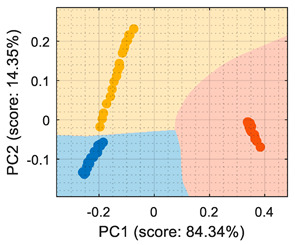
(c) PCA + SVM	100	0	100	0	100	0	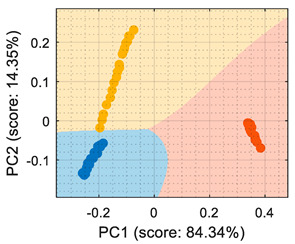
(d) LDA	100	0	100	0	100	0